# Exploring senescence as a modifier of β cell extracellular vesicles in type 1 diabetes

**DOI:** 10.3389/fendo.2024.1422279

**Published:** 2024-08-22

**Authors:** Roozbeh Akbari Motlagh, Jasmine Pipella, Peter J. Thompson

**Affiliations:** ^1^ Department of Physiology and Pathophysiology, Rady Faculty of Health Sciences, University of Manitoba, Winnipeg, MB, Canada; ^2^ Diabetes Research Envisioned and Accomplished in Manitoba (DREAM) Theme, Children’s Hospital Research Institute of Manitoba, Winnipeg, MB, Canada

**Keywords:** type 1 diabetes, cellular senescence, extracellular vesicles, microRNAs, SASP (senescence-associated secretory phenotype)

## Abstract

Type 1 Diabetes (T1D) is a chronic metabolic disease resulting from insulin deficiency due to autoimmune loss of pancreatic β cells. In addition to β cell destruction, it is now accepted that β cell stress and dysfunction, such as senescence, plays a crucial role in the development of the disease. Accumulation of senescent β cells occurs during development of T1D in humans and contributes to the progression of T1D in the nonobese diabetic (NOD) mouse model. Senescent β cells are thought to exacerbate the inflammatory response within the islets by production and secretion of senescence-associated secretory phenotype (SASP). Extracellular vesicles (EVs) from β cells have been shown to carry protein and microRNAs (miRNAs), influencing cellular signaling and may contribute to the development of T1D but it remains to be addressed how senescence impacts β cell EV cargo. In this minireview, we discuss emerging evidence that EV cargo proteins and miRNAs associated with senescence could contribute to the development of T1D and could suggest potential biomarkers and therapeutic targets for the regulation of SASP and elimination of senescent β cells in T1D. Future investigation exploring the intricate relationship between β cell senescence, EVs and miRNAs could pave the way for the development of novel diagnostic techniques and therapeutic interventions.

## 1 Introduction

Type 1 diabetes (T1D) is a chronic metabolic disease resulting from severe destruction of β cells by an autoimmune T-cell-mediated response, manifested by insulin deficiency (1). T1D is a complex disease driven by genetic susceptibility, environmental factors, and epigenetic changes (2) that progresses through three stages. At the first stage, the onset of autoimmunity is accompanied by seropositivity against two or more specific proteins, such as glutamate decarboxylase 65 (GAD65), insulin, insulinoma antigen-2 (IA-2) and zinc transporter 8 (ZnT8) (3). A clinical manifestation of T1D typically occurs months or years before two or more of these autoantibodies are detected (4). Stage 2 is characterized by glucose intolerance, but the patient is otherwise asymptomatic. The third and final stage is marked by the emergence of diabetes symptoms, accompanied by a sustained decline in β cell function for several years (5). Although significant progress has been made in understanding T1D pathogenesis and the clinical implementation of the first FDA-approved therapy for use prior to symptoms onset, Teplizumab (6), a *bona fide* cure has remained elusive and major questions remain about how the disease develops, hindering advancement of therapies for the disease (7, 8).

A growing body of evidence has now shown that β cell dysfunction contributes to T1D. β cell dysfunction in T1D involves various stressed states, including the Unfolded Protein Response (UPR), type I interferon (IFN) response and senescence (9, 10). While investigations into the therapeutic potential of modulating β cell UPR and IFN response have progressed into phase II clinical trials with encouraging results (11–14), preclinical investigations into β cell senescence are still required to pave the way for clinical translation. The interactions between β cells and immune cells during T1D remains a puzzle (15), but exciting efforts have also suggested roles for small secreted extracellular vesicles (EVs) as important conveyors of signaling between β cells and immune cells during the pathogenesis of T1D (16). EVs are a heterogeneous group of nanoparticle-sized membrane-bound cargo carriers secreted from most cell types, containing DNA, RNA, proteins, and metabolites from their host cell. EVs consist of exosomes (30-150 nm) in addition to larger particles termed microvesicles (200-1000 nm) and apoptotic bodies (>1000 nm) (17). Remarkably, cellular senescence has been shown to dramatically increase small EV biogenesis and alter EV RNA and protein cargoes as a component of the senescence-associated secretory phenotype (SASP) (18–20). However, it remains unclear how senescence alters EV biogenesis and cargo in the β cell and what role, if any, senescent β cell EVs may play in T1D development.

In this minireview, we discuss the potential for senescent β cell EVs to contribute to the pathogenesis of T1D. We propose a novel approach to therapy, wherein the utilization of miRNA packaged into therapeutic EVs could target senescent β cells to mitigate their inflammatory effects.

## 2 Relationships between β cell EVs, senescence and miRNAs: implications for development of T1D and novel therapies

### 2.1 β cell EVs and development of T1D

**Figure 1:**
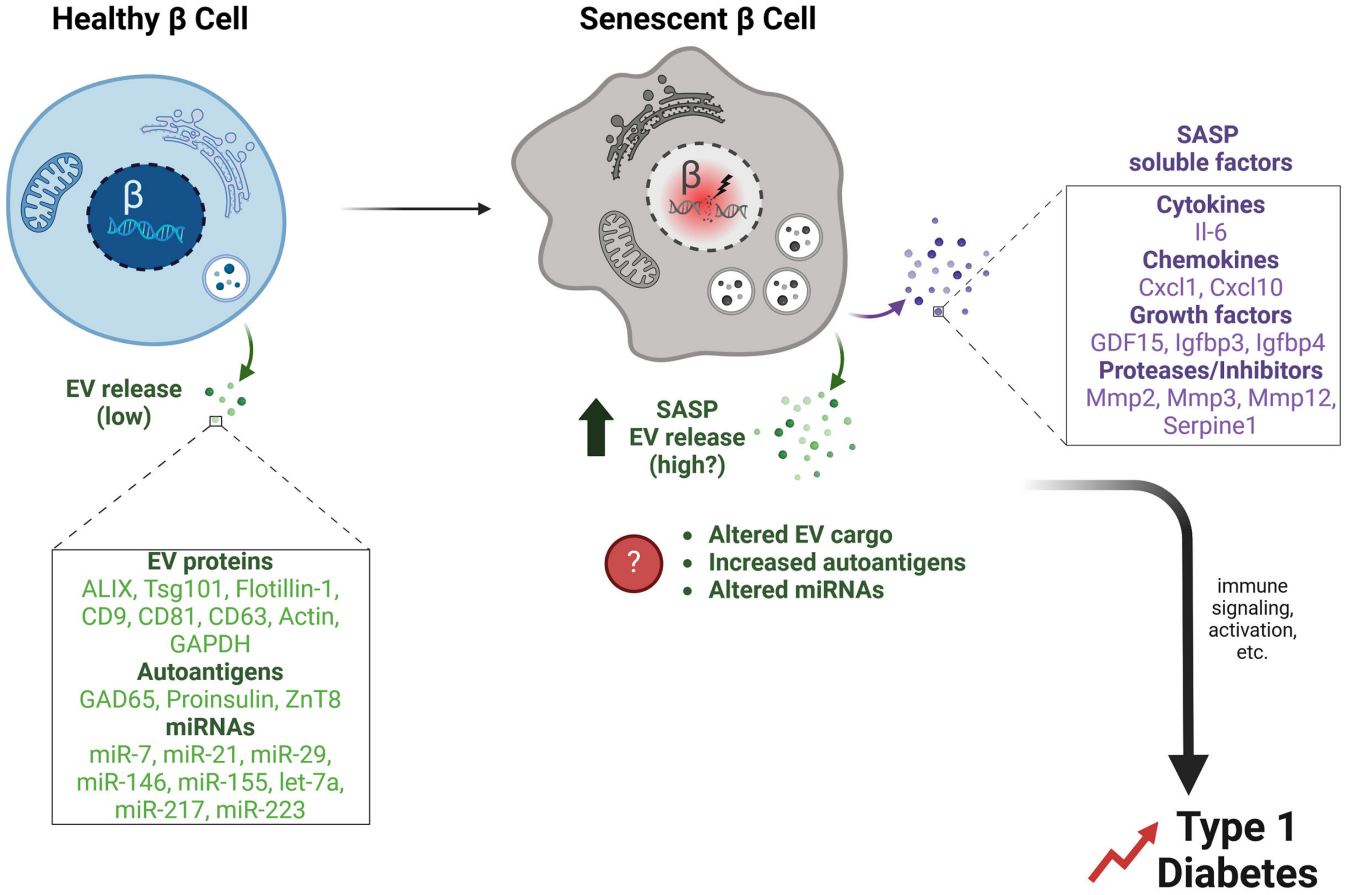
β cell EVs and potential effects of senescence on EV release and cargo. Healthy primary islet β cells release a low quantity of exosomes under typical culture conditions. β cell EVs contain typical EV proteins (Alix, Tsg101, Flotillin-1, CD9, CD81, CD63, Actin, GAPDH), along with autoantigens (GAD65, Proinsulin, ZnT8), and miRNAs (miR-7, miR-21, miR-29, miR-146, miR-155 let-7a, miR-217, miR-223). In contrast, senescent β cells that accumulate during T1D develop a SASP, which we propose involves not only classical SASP factors (SASP soluble factors) but also increased release of EVs (SASP EV release). Senescence may also alter β cell EV cargo, such as increasing the release of autoantigens and changing the miRNA profile. Together these changes in β cell EVs during senescence could facilitate immune signaling and activation events that accelerate the development of T1D.

Early evidence of EVs secretion by pancreatic β cells was reported by Sheng H. and colleagues in 2011, which showed EVs released from the mouse β cell line MIN6 (21). As with many other cell types, β cells produce and secrete mainly the small exosome-sized EVs rather than microvesicles (MVs) as demonstrated by culture studies using ultracentrifugation-based approaches for EV isolation (22) on rodent β cell lines or isolated islets from rodents and humans (23–26). While highly proliferative rodent β cell lines such as MIN6 and the rat β cell line INS-1, showed robust EV secretion, isolated rodent, and human primary islets, which generally do not proliferate, secreted few EVs in comparison, suggesting that EV release may be associated with β cell proliferation. Proteomic analysis of β cell EVs from mouse and human sources showed that they contain canonical exosomal markers, including endosomal sorting complex required for transport (ESCRT) machinery (Alix, Flotillin-1, Tsg101), tetraspanins (CD9, CD81, CD63), as well as housekeeping proteins (GAPDH, Actin) (**Figure 1**). Interestingly, primary mouse and human islet β cell EVs also contained putative autoantigens, including GAD65, proinsulin and ZnT8, which were shown to be released in a caspase-independent manner and at higher rates during proinflammatory cytokine exposure (23, 24) (**Figure 1**). Notably in regards to tetraspannin markers, a recent study showed that CD63 levels discriminate between two different β cell subsets in mice and humans, where CD63^High^ β cells had higher metabolic and functional capacity compared with CD63^Low^ (27), suggesting possible differences in EV markers or biogenesis associated with β cell function. However, further studies to test this hypothesis are clearly warranted.

The observation that β cell EVs contain autoantigens has raised the intriguing possibility that β cell EVs convey autoantigens directly to the immune system to activate and drive autoimmunity. Indeed, one study found that human islet EVs are internalized by monocytes and B cells, provoking T cell activation and memory responses in peripheral blood mononuclear cells (PBMCs) from T1D donors (28). EVs containing GAD65 triggered the production of anti-GAD antibodies from B cells (28) providing an explanation as to how β cell autoantigens are being sampled and provoke immune responses during T1D progression. In other work, it was demonstrated that the proinflammatory chemokine CXCL10 is enriched on the surface of stressed β cell EVs, promoting β cell dysfunction via paracrine effects in addition to immune cell chemotaxis (26). Inhibition of small EV release rescued defects in proinflammatory cytokine-stressed primary islet function and reduced macrophage and CD8^+^ T cell infiltration (26), suggesting a role for stressed β EVs in promoting islet dysfunction and immune recruitment.

Despite these advances and the insights learned from tissue culture studies, the question of how and whether β cell EV release actually contributes to autoimmunity *in vivo* during the development of T1D has not been addressed. In particular, it remains a major challenge in the field to specifically determine the cell type of origin for isolated EV populations from blood or even islets in culture, since EV populations are a heterogeneous mixture produced by multiple cell types and cell-type specific surface markers on EVs for β cells have not yet been identified. To tackle this challenge, recently developed conditional reporter mouse models could be used to examine the impact of senescence on β cell EVs Specifically, a tissue-specific EV reporter mouse model (e.g., lox-STOP-lox-CD9-GFP) could be employed, where the conditional activation occurs in β cells (e.g., by crossing with an Ins1-Cre driver line).These allow for tissue-specific fluorescent protein tagging of EVs, such as the Transgenic inducible GFP extracellular vesicle reporter (TIGER) mouse which uses a CD9-GFP (29), a truncated CD9-GFP EV conditional reporter that allows for affinity isolation (30), or the CD63-GFP conditional reporter mouse models (31, 32). Senescent beta cells could then be ablated by treating the animals with senolytics (compounds the trigger apoptosis preferentially in senescent cells (33), and comparing beta cell EV cargo from control and senolytic-treated animal islets. Given that β cell EVs naturally express these tetraspanin markers, these mouse models could provide key tools for addressing how β cells release EVs *in vivo*, elucidating the trafficking of these EVs and the impact of stress on β cell EV release during the development of T1D.

### 2.2 The role of senescent β cells and the potential role of senescence-derived EVs in T1D

Cellular senescence is a stress response leading to various hallmark phenotypes including an irreversible cell cycle arrest, resistance to apoptosis, metabolic adaptation and acquisition of a SASP (34). Although senescence is beneficial in certain contexts such as during embryogenesis and wound healing, when senescent cells are not efficiently removed by the immune system, their accumulation leads to chronic inflammation and tissue dysfunction (35). Senescent β cells that accumulate during T1D development in the NOD autoimmune diabetes mouse model and human donor pancreas tissue exhibit the major hallmarks of senescence in other cell types. These include a DNA damage response (DDR), apoptosis resistance, the lysosomal senescence-associated β-galactosidase (SA-βgal) and a SASP (Cha et al., 2023) although the precise triggers responsible for the induction of senescence in β cells during T1D are still unknown. Senescent β cells in NOD mice upregulate the prosurvival protein Bcl-2, rendering them sensitive to Bcl-2 inhibitor senolytics, which can selectively trigger apoptosis in this subset of β cells (36).

Senescent cells activate the release of a proinflammatory secretome known as SASP, which in β cells comprises cytokines and chemokines (e.g., IL-6, Cxcl10, Ccl2, Ccl4, Cxcl1, Cxcl8), matrix metalloproteinases and inhibitors (MMP-2, MMP-3, MMP-12, Serpine1), and growth factors (Gdf15/GDF15, Igfbp3, Igfbp4/IGFBP4) (15) (**Figure 1**). The various biological activities elicited by the SASP are intimately connected to the microenvironment in which senescent cells reside. In β cells, SASP can also exert detrimental paracrine effects on adjacent non-senescent healthy β cells, such as triggering senescence (36–38). Additionally, the induction of SASP in cultured human islets causes changes in glucagon secretion from α cells, implying that the SASP originating from β cells can have an impact on the normal functioning of α cells (39). Suppression of SASP from senescent β cells in NOD mice protects against diabetes (40), supporting a deleterious role for SASP in T1D development.

In addition to the secretion of soluble immunogenic proteins, recent studies have also shown that SASP involves increased secretion of EVs and altered cargo compared to the EVs released from non-senescent cells (19, 20). Notably, senescent cells are more sensitive to inhibition of EV release compared with non-senescent cells (41), and loss of EV release machinery proteins can accelerate senescence onset, leading to speculation that EV release may provide an adaptive mechanism for removal of unwanted cargo in senescent cells (42). Senescence also alters EV miRNA cargo (43, 44). However, it remains to be determined how senescence alters β cell EV release and cargo (**Figure 1**). Although proteomic studies have been carried out to identify β cell SASP factors, EVs were not specifically isolated in those studies (45). Similarly, while it was recently shown that miRNAs have coding motifs that direct their sorting into exosomes (46), the extent to which this mechanism operates to direct miRNA sorting in β cell EVs in the context of senescence remains an open question. We postulate that, aside from its effects through the direct release of soluble paracrine factors, β cell SASP could also cause detrimental impacts on neighboring or distant cells through increased release of EVs with modified protein and/or miRNA cargo (**Figure 1**). For instance, senescence may increase autoantigen cargo or lead to the export of immune-activating miRNAs. The broader scope of SASP influence is facilitated by the transmission of EVs, allowing it to potentially affect cells that are distant from islets.

### 2.3 β cell EV miRNAs and impact of senescence on EV miRNA

In addition to protein cargo, miRNAs are also enriched in β cell EVs. miRNAs are non-coding RNAs 21-23 nucleotides long, regulate gene expression post-transcriptionally. They inhibit mRNA translation or reduce mRNA stability, playing key roles in genetic regulation and various biological processes (47). Cataloging more than 2500 miRNAs in humans has revealed their significant role in regulating around 60% of protein-coding genes within the genome. While miRNAs are predominantly generated and function within cells, recent studies have shed light on their ability to be secreted outside the cell in micro-vesicles or exosomes. This intriguing finding has expanded our understanding of miRNAs, revealing their capacity to transmit their regulatory functions to other cell types. β cell EVs contain: miR-7, miR-21, miR-29, miR-146a, miR-223, let-7a, miR-217 and miR-155 (25, 48–52) (**Figure 1**). Interestingly, some of the targets for these miRNAs include genes involved in senescence/SASP, including *Bcl2 CXCR2*, *MMP2*, *MMP9*, *Cdkn2a* (encoding p16^Ink4a^), *TP53, TNF*, and *IL6*. For example, miR-21 increases in abundance during proinflammatory cytokine exposure in the MIN6 β cell line and primary isolated mouse and human islets (25). miR-21 increases β cell susceptibility to apoptosis by downregulating *Bcl2* mRNA levels (53), and overexpression of miR-21 precursor pre-miRNA in β cells leads to defects in glucose-stimulated insulin secretion (54), suggesting a novel link between proinflammatory conditions and impairment of β cell function via miRNA expression.

Upon entering senescence, the expression patterns of miRNAs undergo notable alteration (55). This alteration in miRNA expression is also manifested in the composition of EVs (55). Alibhai et al. revealed that in aging mice, the levels of miR-146a, miR-21, miR-223, and let-7a in circulating EVs in plasma increased, indicating a correlation with modifications in immune function (56). Another study observed that EVs released by replicative senescent human umbilical vein endothelial cells, enriched with elevated levels of miR-21-5p and miR-217, were observed to reduce proliferation and promote senescence in neighboring endothelial cells (57). Salama et al. have observed that miR-29 in β cell-derived exosomes induced TNFa, and IL-6 in splenocytes of NOD mice (49). Up-regulation of miR-29 correlated with DNA damage response, increased levels of the DNA damage marker γ-H2A.X, and accumulation of SA-βGal (58). miR-29 was also upregulated in aged mice (59). Moreover, EVs containing miR-29 were considered controls for aging and the induction of an inflammatory environment in white adipose tissue (60). Davis et al. utilized a miRNA array to showcase a variation in miRNA expression within EVs obtained from the bones of aged and youthful mice. The EVs originating from the bones of older mice exhibited a distinct elevation in the levels of miR-183-5p. Moreover, the overexpression of a miR-183-5p mimic exhibited the ability to stimulate senescence and hinder the proliferation and differentiation of Bone Marrow Mesenchymal Stromal Cells (BMSCs) (61). Thus, it is apparent that in pathological situations, the isolated EVs can have cell type specific effects in the microenvironment. Although the impact of senescence has not been studied on islet EVs, a recent preprint reported that isolated EVs from human islets treated with inflammatory cytokines led to the upregulation of miR-155-5p (52). These findings were in line with the analysis of EVs plasma derived from children with recent T1D and autoantibody-positivity (52) suggesting inflammatory stress could provoke β cell miR-155 EV release into circulation.

In summary, despite the considerable number of studies conducted on senescent cell EVs and their miRNA cargo and impacts of inflammatory stress on β cell EVs, there remains a gap in understanding senescent β cells and identifying EVs produced by senescent β cells. To address this question, genetic tools to disrupt the β cell senescence program *in vivo* or ablate p21-expressing senescent cells, such as in the p21-Cre mouse (62) or the p21 promoter-driven suicide gene mouse model (p21-ATTAC) (63) could be utilized. These models would allow for ablation of senescent beta cells, thereby also eliminating their potentially altered EVs. In addition, now that reliable approaches for inducing senescence in mouse and human β cells *in vitro* have been established by us (64) and others (45), they provide suitable culture models to investigate the impact of senescence on β cell EV release and cargo. These strategies will enable the isolation of EVs from control versus senescent β cells for transcriptomic analyses to gain a deeper comprehension of how senescent β cell EV miRNAs could promote the progression of autoimmune β cell loss in the islet microenvironment.

The heterogeneity in EVs complicates the task of isolating and identifying specific EV subtypes (e.g., islets-derived EVs) amid other non-target EVs circulating in the body. Therefore, the exploration of novel biomarkers on the surface of EVs is of great interest. The application of flow cytometry has been used to address the heterogeneity of senescent cell EVs, both *in vitro* and *in vivo.* Meng et al. (65) identified the proteins present on extracellular vesicles associated with senescence (S-EVs) across various senescence models. Their findings revealed that the proteins DPP4, ANXA1, ANXA6, S10AB, AT1A1, and EPHB2 were predominantly present on the surface of EVs from senescent cells. DPP4 is distinguished by its unique capability to impede EV uptake by proliferating cells. Furthermore, it was noted that there was a marked increase in DPP4-rich EVs in the blood of women with gestational diabetes mellitus (GDM) (66). DDP4-rich EVs were also reported in the urine of patients with diabetic kidney disease (67). This raises the question of whether DPP-4-bearing EVs could be used as a unique biomarker for applying flow cytometry to identify senescent β cell-derived EVs *in vivo*. Future efforts in this area could use a screening approach for plasma membrane proteins that are selectively expressed on the surface of EVs from senescent β cells. By performing proteomics analysis to identify new potential markers of β cell senescence found on the surface of EVs, the effectiveness of drugs targeting senescent β cells could be more effectively evaluated.

### 2.4 Therapeutic EVs to improve targeting of senescent β cells in T1D

Pharmacological elimination of senescent cells predominantly hinges on two specific types of compounds known as senolytics and senomorphics (68). Senolytic compounds are designed to specifically target and eliminate senescent cells while minimizing any impact on the proliferating and healthy cells, while senomorphics act to modify senescent cell phenotypes in a beneficial manner (68, 69). While there are not yet available tools to specifically manipulate the EV release from senescent cells (while not impacting EV release generally from other cell types) both senolytics and senomorphics could mitigate senescent β cell EV release, since senolytics would eliminate senescent β cells themselves along with their EVs and senomorphics could suppress SASP which also involves altered EV release. Most efforts have focused on use of senolytic compounds which have been extensively tested both *in vitro* and *in vivo*, including inhibitors targeting the BCL-2 family such as ABT-263 and ABT-737 (68). Recently clinical trials suggest the senolytic cocktail dasatinib and quercetin (D+Q) is safe and promising for treating senescence-associated diseases in humans (70–72). While senolytics have not reached the clinic for treating T1D, preclinical studies support the positive influence of senolytic compounds ABT-199 and ABT-737 in depleting senescent β cells in NOD mice, which protected against T1D (36). However, senolytic compounds are administered systemically and can have off-target effects. Senolytics also display significant variability in efficacy across various cell lines and tissues, making it problematic to identify a single senolytic as a commonly implemented method for eliminating senescent cells *in vivo* (69).

**Figure 2:**
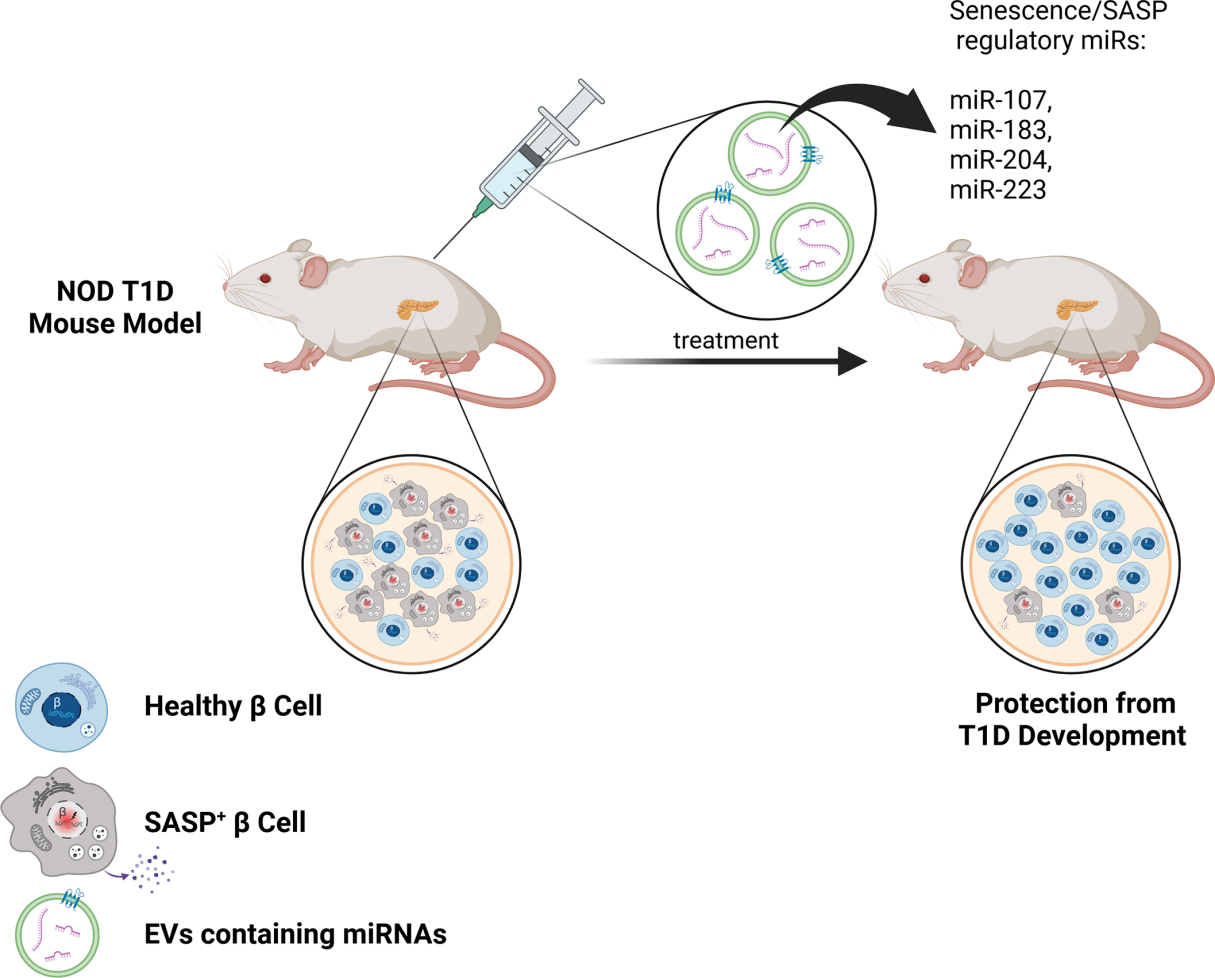
Therapeutic EV delivery of senotherapeutic miRNAs for improved targeting of SASP in β cells during T1D. The use of specially designed EVs with surface molecules to target β cells (such as GLP1R agonists) and harboring miRNAs to suppress SASP (such as miR-107, miR-183, miR-204, miR-223) could be a powerful approach for improved targeting of SASP^+^ β cells and sparing healthy β cells *in vivo* during the development of T1D. This approach could downregulate SASP preferentially in β cells, thereby reducing off-target effects of other senotherapeutics and protecting against T1D.

Another approach to targeting senescence is using senomorphics to directly target and inhibit the SASP process. Suppression of β cell SASP using Bromodomain Extra-Terminal domain (BET) protein inhibitor iBET-762 protects against T1D in NOD mice (40). However, BET proteins are ubiquitously expressed and employed in a variety of inflammatory and housekeeping processes unrelated to SASP, making it highly problematic to adapt this senomorphic approach to T1D in humans. On the other hand, miRNAs may provide an under-appreciated mechanism to regulate and modify the SASP. Numerous studies have highlighted the role of miRNAs in the regulation of senescence signaling pathways, and specifically in controlling SASP (47, 73). For example, Qi et al. indicated that miR-204 suppresses IL-6, IL-18, and TNF-α by upregulation of Bcl-2 and SIRT1 in rats with diabetic retinopathy (74). Studies have demonstrated that miR-107 triggers apoptosis and downregulates the expression of CXCR2, MMP-2, and MMP-9, leading to a decrease in SASP secretion (75). Furthermore, miRNAs are involved in the modulation of senescence through the key cell cycle arrest regulators p16^Ink4a^ and p53 (73). Indeed, miR-24 effectively hinders replicative senescence by repressing *Cdkn2a* expression through binding to the 3′UTR of its mRNA (76). EVs have been proposed as next generation treatment alternatives and designer nanoparticles to transport medications (77). For example, EV-delivered miRNAs have reached clinical trials for cancer therapy (78). Therefore, EVs may present a natural, cell/tissue-derived avenue for efficient transport of senolytic or senomorphic miRNAs to improve targeting efficiency of senescent β cells in T1D, acknowledging that other cargo of β cell EVs may play roles in T1D as well (**Figure 2**). EVs may have lower immunogenicity as compared with other delivery reagents, as shown in recent studies where no toxic effects were observed in mice after three weeks of treatment with EVs from the human embryonic kidney cell line HEK293T (77). EVs also may provide a suitable barrier to prevent the degradation of therapeutic miRNAs. Surface proteins on EVs may act like distinct barcodes, helping delivered EVs to reach their intended targets *in vivo*. In this regard, in previous studies, engineered EVs with surface localization of a β cell protein p88 fused to EV protein lactoadherin showed increased uptake to the pancreas upon intravenous administration in mice (79). In the future, it will be crucial to identify and characterize miRNAs that suppress SASP in β cells. The use of targeted EV surface proteins (such as p88 or ligands for GLP1R that is enriched on β cells) along with loading of miRNA cargo to inhibit SASP could provide precision suppression of senescent β cell SASP *in vivo*, while reducing the impacts of off-target effects associated with systemic senomorphic use (**Figure 2**).

## 3 Conclusion

In conclusion, current evidence suggests that β cell EVs could play a critical role in T1D progression. However, the specific models have been lacking to definitively address this question *in vivo*. The advent of conditional EV reporter mouse models has furnished the islet biology field with tools that will undoubtedly provide insights into how β cell EVs contribute to T1D pathogenesis. We propose that β cell senescence alters EV production along with the protein and miRNA cargo in a manner that accelerates T1D (**Figure 1**). As methods to engineer EVs continue to improve, we also anticipate that EV technology will enable the use of surface ligands with high β cell/islet specificity and these tools could be deployed to improve upon systemically administered therapeutics for targeting senescent β cells (**Figure 2**). As EVs have already been used to effectively package and deliver miRNA cargo in clinical trials, we suggest that a similar approach applied to miRNAs that regulate SASP, could prove highly effective for limiting the deleterious impact of accumulated senescent β cells in diabetes.
